# Delineating the expanding phenotype associated with *SCAPER* gene mutation

**DOI:** 10.1002/ajmg.a.61202

**Published:** 2019-06-13

**Authors:** James Fasham, Gavin Arno, Siying Lin, Mingchu Xu, Keren J. Carss, Sarah Hull, Amelia Lane, Anthony G. Robson, Olivia Wenger, Jay E. Self, Gaurav V. Harlalka, Claire G. Salter, Lynn Schema, Timothy J. Moss, Michael E. Cheetham, Anthony T. Moore, F. Lucy Raymond, Rui Chen, Emma L. Baple, Andrew R. Webster, Andrew H. Crosby, NIHR Bioresource Rare Diseases Consortium

**Affiliations:** ^1^ Medical Research, RILD Wellcome Wolfson Centre University of Exeter Medical School, Royal Devon and Exeter NHS Foundation Trust Exeter United Kingdom; ^2^ Peninsula Clinical Genetics Service Royal Devon and Exeter Hospital (Heavitree) Exeter United Kingdom; ^3^ UCL Institute of Ophthalmology University College London London United Kingdom; ^4^ Moorfields Eye Hospital London United Kingdom; ^5^ Department of Molecular and Human Genetics Baylor College of Medicine Houston Texas; ^6^ Human Genome Sequencing Center Baylor College of Medicine Houston Texas; ^7^ Department of Haematology NHS Blood and Transplant Centre, University of Cambridge Cambridge United Kingdom; ^8^ NIHR BioResource – Rare Diseases Cambridge University Hospitals NHS Foundation Trust Cambridge United Kingdom; ^9^ New Leaf Center Clinic for Special Children Mount Eaton Ohio; ^10^ Clinical and Experimental Sciences, Faculty of Medicine University of Southampton Southampton United Kingdom; ^11^ Division of Genetics and Metabolism University of Minnesota Medical Center – Fairview Minneapolis Minnesota; ^12^ Division of Genetics and Metabolism, Department of Pediatrics University of Minnesota Minneapolis Minnesota; ^13^ Ophthalmology Department, UCSF School of Medicine Koret Vision Centre San Francisco California; ^14^ Department of Medical Genetics, Cambridge Institute for Medical Research University of Cambridge Cambridge United Kingdom

**Keywords:** Brachydactyly, CCNA2–CDK2, Intellectual disability, Retinitis pigmentosa, SCAPER

1

A potential role for the cyclin A2–cyclin‐dependent kinase 2 complex regulator S‐phase cyclin A‐associated protein residing in the endoplasmic reticulum (SCAPER) in human disease was first suggested by Najmabadi et al. (2011), who identified a candidate homozygous frameshift *SCAPER* variant as the cause of nonsyndromic intellectual disability (ID) in a small Iranian family. We subsequently reported a single patient with biallelic loss of function (LOF) *SCAPER* variants associated with retinal disease (Carss et al., 2017). Biallelic LOF variants have since been associated with ID with or without retinitis pigmentosa (RP) in seven individuals from five families from Spain, Israel, and Iran (Hu et al., 2018; Tatour et al., 2017); in one individual from a Jordanian Arab family, a homozygous *SCAPER* gene variant was identified as the cause of nonsyndromic RP (Jauregui et al., 2019). More recently, Wormser et al. (2019) described a *SCAPER* gene variant associated with a Bardet–Biedl syndrome (BBS)‐like presentation comprising of ID, RP, short stature, obesity, and brachydactyly in eight individuals from two consanguineous Bedouin families belonging to the same tribe in southern Israel, alongside preliminary functional studies suggesting a possible role for SCAPER in ciliary dynamics and disassembly. In the current study, we describe clinical and genetic findings, including seven novel *SCAPER* variants, in six individuals of Amish, Caucasian, and South Asian descent. Together with our molecular data, our comprehensive phenotypic assessments enable a more detailed clinical comparison to be drawn between the patient cohort described here (including previously published individual G001284; Patient 3 in this study, (Carss et al., 2017) with the 17 individuals in whom *SCAPER* variants were recently defined (Hu et al., 2018; Jauregui et al., 2019; Najmabadi et al., 2011; Tatour et al., 2017; Wormser et al., 2019), permitting a more precise definition of the clinical phenotype arising from pathogenic *SCAPER* variation.

Samples were taken with informed consent (study approved by the Ethics Committee of Akron Children's Hospital, Moorfields Eye Hospital and Baylor College of Medicine, in compliance with the Declaration of Helsinki) for deoxyribonucleic acid (DNA) extraction. Single nucleotide polymorphism (SNP) genotyping was performed (Patients 1 and 2) using the HumanCytoSNP‐12 v2.1 beadchip array (Illumina, Cambridge, UK). In Patients 1 and 3–5, whole exome or whole genome sequencing (WES or WGS), variant alignment, calling, filtering, and prioritization was performed as previously described (Carss et al., 2017; Rawlins et al., 2019; Xu et al., 2015). Allele‐specific primers were designed using Primer3 web software to evaluate segregation of the candidate *SCAPER* gene variants identified via dideoxy sequencing. Patient 6 underwent WES at GeneDx and was identified via GeneMatcher (Sobreira, Schiettecatte, Valle, & Hamosh, 2015) as part of the Matchmaker Exchange Repositories (Philippakis et al., 2015). All variants identified in the study have been submitted to ClinVar (https://www.ncbi.nlm.nih.gov/clinvar/).

Patients 1 and 2 are Ohio Amish siblings. Candidate variants identified through WES of DNA from Patient 1 were cross‐referenced with regions of autozygosity common to both affected siblings, identified through whole genome SNP genotyping. This identified only a single plausible candidate variant, located within the largest (18 Mb) shared region of autozygosity on chromosome 15 (rs1509805–rs4243078; chr15(GRCh38):g. 60281446‐78374545), a novel homozygous duplication in Exon 18 of the *SCAPER* gene, predicted to result in a frameshift (NM_020843.2: c.2236dupA, p.(Ile746Asnfs*6) Chr15(GRCh38):g.76705914dupT; Figure 1). Dideoxy sequencing confirmed the presence and co‐segregation of this variant in both siblings. This variant was detected in heterozygous form in five unrelated individuals in a database of 116 regional Amish controls, corresponding to an estimated allele frequency of ~0.04, not uncommon for founder mutations within this population. WES/WGS performed in Patients 3–6, identified compound heterozygous *SCAPER* variants; c.1116delT, p.(Val373Serfs*21) (Chr15(GRCh38):g.76771874delA) and c.2179C>T, p.(Arg727*) (Chr15(GRCh38):g. 76705971G>A) in Patient 3, c.1495+1G>A (Chr15(GRCh38):g.76765562C>T) and c.3224delC, p.(Pro1075Glnfs*11) (Chr15(GRCh38):g.76434165delG) in Patient 4, c.829C>T, p.(Arg277*) (Chr15(GRCh38):g. 76775061G>A) and c.3707_3708delCT, p.(Ser1236Tyrfs*28) (Chr15(GRCh38):g.76376309_ 76376310delAG) in Patient 5, and c.2377C>T, p.(Gln793*) (Chr15(GRCh38):g.76702873G>A) and c.2166‐3C>G (Chr15(GRCh38):g.76705987G>C) in Patient 6. The *SCAPER* variants in each of these patients were confirmed to be biallelic by familial segregation analysis using dideoxy sequencing. None of these variants are present in the genome aggregation (gnomAD) or 1,000 genomes databases and those in Patients 1, 2, and 4–6 are novel.

**Figure 1:**
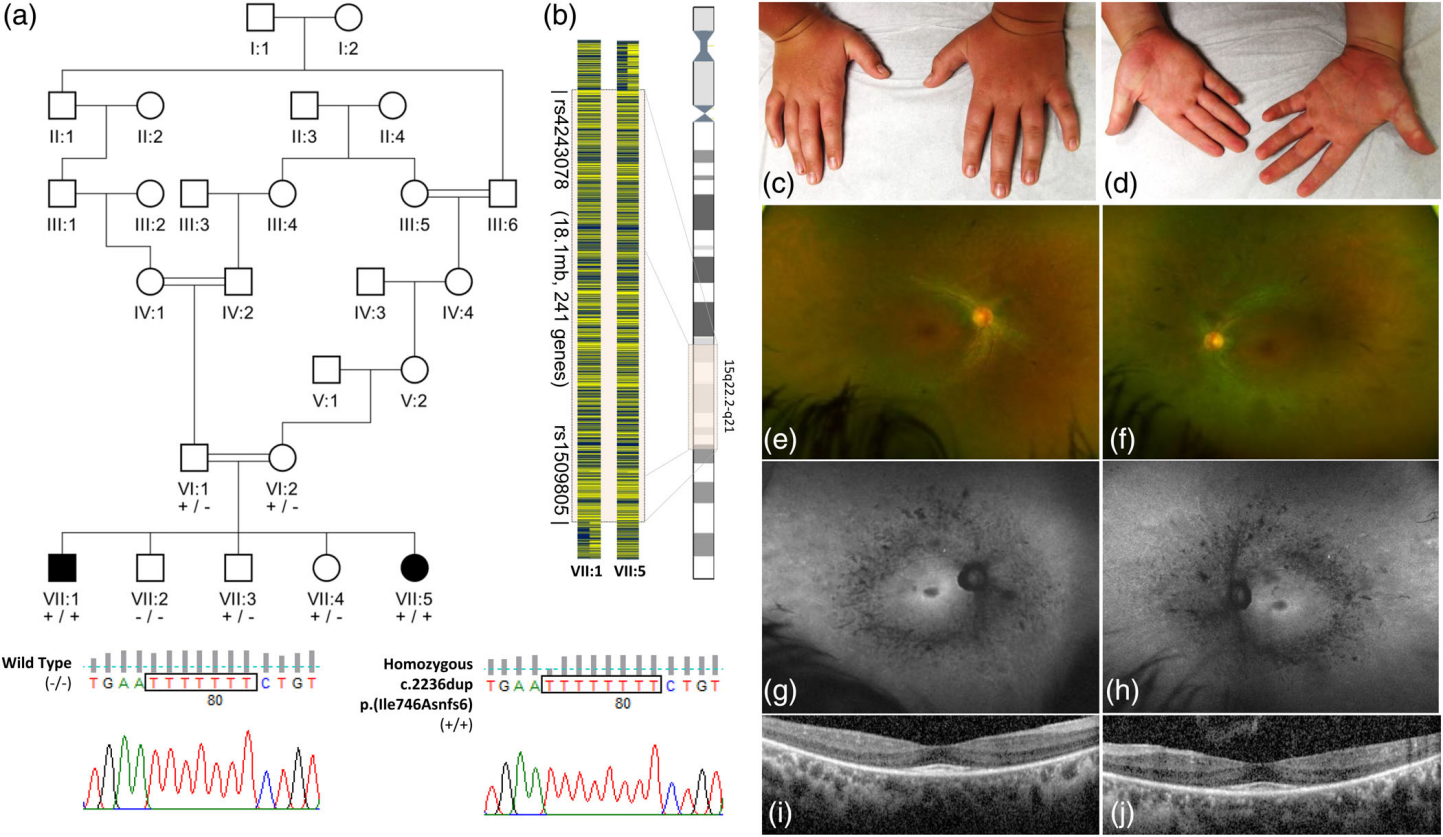
(a) Simplified pedigree of the Amish family investigated, with electropherograms showing the *SCAPER* c.2236dupT sequence variant in all affected and unaffected individuals in generations VI and VII (black arrow identifies the duplicated nucleotide). (b) Pictorial representation of the single nucleotide polymorphism (SNP) genotypes across the ~18.1 Mb chromosome 15q21‐22 region identified in this family. (c–j) Clinical features of *SCAPER* syndrome patients. (c, d) Brachydactyly, camptodactyly, and proximally placed thumbs identified on examination of patient 1. (e, f) ocular imaging and investigations from patient 3 illustrating features of RP (e: Right eye, f: Left eye) fundus photograph (Optos plc, Dunfermline, UK) showing optic disc pallor, attenuated retinal vessels and mid‐peripheral bone spicule pigmentation (g: Right eye, h: Left eye) FAF imaging showing mid‐peripheral hypoautofluorescence with a central ring of hyperautofluorescence demarcating the surviving outer retinal structures. (i: Right eye, j: Left eye) optical coherence tomography (Spectralis‐OCT, Heidelberg Engineering, Heidelberg, Germany) of the central retina demonstrating loss of photoreceptor outer segments with retained central macular structure corresponding to FAF findings. FAF, fundus autofluorescence; OCT, optical coherence tomography; SCAPER, S‐phase cyclin A‐associated protein residing in the endoplasmic reticulum

Table 1 summarizes the core phenotypical features of individuals not previously reported, aged between 18 months and 31 years (Patients 1, 2, and 4–6), provides additional clinical details for Patient 3 (Carss et al., 2017), and compares these to the clinical features of all *SCAPER* syndrome patients described to date. ID and developmental delay was present in all six affected individuals, and four patients also exhibited hyperactivity and attention deficit hyperactivity disorder (ADHD). Autism and dyspraxia were each noted in one individual. Neuroimaging performed in Patients 1, 3, 5, and 6 revealed no abnormalities. Additional dysmorphic features noted in both Amish siblings (Patients 1 and 2) included inverted nipples, brachydactyly, camptodactyly, proximally placed thumbs (Figure 1), and a characteristic facial appearance with frontal bossing and almond‐shaped eyes; growth parameters were all normal. Patients 1, 3, and 4–6 all presented between the ages of 10–23 with a reduction in night vision and visual field deficits; Patient 2 (18 months) described no visual symptoms at the time of presentation. Fundus examination in Patients 3–6 revealed findings typical of RP including optic disc pallor, attenuated retinal vessels and intraretinal mid‐peripheral bone‐spicule pigmentation, and loss of photoreceptor outer segments with retained central macular structure on optical coherence tomography imaging (Figure 1; Table S1). Additional variable ocular features described in some patients with *SCAPER* syndrome include cataracts (in two individuals) and myopia and keratoconus in one individual each.

**Table 1:** A comparison of clinical findings of all affected individuals with biallelic pathogenic *SCAPER* variants

	Genotype	Ethnicity	Sex	Age (years)^a^	Weight (kg, SDS)	Height (cm, SDS)	OFC (cm, SDS)	BMI (SDS)	Walked (months)	Speech delay	ID	Behavior issues	Abnormal neuroimaging	RP	Brachydactyly	Other clinical findings
Najmabadi	p.(Tyr118fs*)/p.(Tyr118fs*)	Iran	NA	NA	NA	NA	NA	NA	NA	NA	✓	NA	NA	NA	NA	NA
Tatour (A:II:1)	c.2023‐2A>G/c.2023‐2A>G	Arab	F	24	NA	NA	NA	NA	Normal	NA	Mild (IQ 64)	ADHD	MRI: Normal	✓	NA	Nil
Tatour (A:II:2)	c.2023‐2A>G/c.2023‐2A>G	Arab	F	23	NA	NA	NA	NA	Normal	NA	Mild (IQ 56)	ADHD	NA	✓	NA	Nil
Tatour (B:II:1)	p.(Ile991fs*)/p.(Ile991fs*)	Spanish	F	34	NA	NA	NA	NA	24	NA	Mod	None reported	CT: Normal	✓	NA	Alopecia areata
Tatour (C:II:4)	p.(Glu620del)/p.(Ser1219Asn)	Spanish	M	15	NA	NA	NA	NA	Delayed	NA	✓	None reported	NA	✓	NA	NA
Hu (family 166; 3 individuals)	p.(Arg120*)/p.(Arg120*)	Baloch	NA	NA	NA	NA	Normal	NA	NA	NA	✓	NA	NA	NA	NA	NA
Jauregui	c.2023‐2A>G/c.2023‐2A>G	Arab	M	11	NA	NA	NA	NA	NA	NA	No	No	NP	✓	NA	NA
Wormser (P1:V5)	p.(Leu936*)/p.(Leu936*)	Bedouin	F	34	78 (+1.9)	145 (−3.1)	Not reduced	37.1 (+3.1)	NA	✓	Mod	NA	NP	✓	✓	Genu valgum/genu varum
Wormser (P1:V6)	p.(Leu936*)/p.(Leu936*)	Bedouin	M	28	78 (+0.7)	157 (−3.1)	Not reduced	31.6 (+2.3)	NA	✓	Mod	NA	NP	✓	✓	Genu valgum/genu varum
Wormser (P1:V7)	p.(Leu936*)/p.(Leu936*)	Bedouin	M	24	98 (+2.2)	163 (−2.2)	Not reduced	36.9 (+3.0)	NA	✓	Mod	NA	NP	✓	✓	Genu valgum/genu varum
Wormser (P1:V8)	p.(Leu936*)/p.(Leu936*)	Bedouin	M	17	92 (+2.2)	155 (−2.9)	Not reduced	38.3 (+3.3)	NA	✓	Mod	NA	NP	✓	✓	Genu valgum/genu varum
Wormser (P2:III1)	p.(Leu936*)/p.(Leu936*)	Bedouin	F	48	86.6 (+2.5)	146 (−3.0)	Not reduced	40.6 (+3.5)	NA	✓	Sev	NA	NP	✓	✓	Nil
Wormser (P2:III2)	p.(Leu936*)/p.(Leu936*)	Bedouin	F	47	62 (+0.4)	149 (−2.5)	Not reduced	27.9 (+1.6)	NA	✓	Sev	NA	NP	✓	✓	Genu valgum/genu varum
Wormser (P2:III7)	p.(Leu936*)/p.(Leu936*)	Bedouin	F	29	57.8 (+0.1)	132 (−5.2)	Not reduced	33.2 (+2.8)	NA	✓	Sev	NA	NP	✓	✓	Nil
Wormser (P2:IV1)	p.(Leu936*)/p.(Leu936*)	Bedouin	M	10	29.5 (−0.38)	129 (−1.5)	Not reduced	17.7 (+0.7)	NA	✓	Mod	ADHD	MRI: abnormal^b^	Suspected	✓	Genu valgum/genu varum
Patient 1	p.(Ile746fs*)/p.(Ile746fs*)	Amish	M	13.7	68.9 (+1.9)	166.3 (+0.7)	56.4 (+0.39)	24.9 (+2.0)	24	✓	Mod	Hyperactivity	MRI: Normal	No	✓	Proximally placed thumbs. Short fifth fingers, pes planus, frontal bossing, almond‐shaped eyes, and inverted nipples
Patient 2	p.(Ile746fs*)/p.(Ile746fs*)	Amish	F	1.5	8.6 (−2.2)	78.5 (−0.7)	47 (−0.92)	14 (−2.5)	22	✓	Mild	Hyperactivity	NP	NA (age)	✓	Proximally placed thumbs, short fifth fingers, pes planus, frontal bossing, almond‐shaped eyes, and inverted nipples
Patient 3^c^ (GC17206)	p.(Arg727*)/p.(Val373fs*)	South Asian	F	28	25th centile	3rd centile	NA	NA	11	✓	Mod	ADHD, autism, and self‐harm	MRI: Normal	✓	NA	Nil
Patient 4 (GC15572)	c.1495+1G>A/p.(Pro1075fs*)	Caucasian	F	31	NA	NA	57 (95th centile)	NA	15	✓	Mild	Dyspraxia	NP	✓	NA	Nil
Patient 5	p.(Arg277*)/p.(Ser1236fs*)	NA (United States)	F	17	NA	NA	NA	Obese	NA	NA	✓	NA	MRI: Normal	✓	NA	Nil
Patient 6	p.(Gln793*)/c.2166‐3C>G	NA (United States)	F	24	63.6 (+0.6)	162.6 (−0.2)	NA	24 (+0.63)	15–18	Yes	Mild (IQ 50–60s)	ADHD	MRI: Normal	✓	NA	Moderate eczema with severe skin‐picking behavior
Summary								8/12 obese		12/12	20/21	8/11	All normal	16/17	10/10	

Abbreviations: ADHD, attention‐deficit hyperactivity disorder; BMI, body mass index; CT, computerized tomography; F, female; ID, intellectual disability; IQ, intelligence quotient (Wechsler Adult Intelligence Scale); M, male; Mod, moderate; MRI, magnetic resonance imaging; NA, not available; NP, not performed; OFC, occipitofrontal circumference; RP, retinitis pigmentosa; SDS, standard deviation scores; Sev, severe.

*Note*. Adults with a BMI >25 are classified as overweight, those >30 are classified as obese; the ✓ symbol indicates the presence of a feature in an affected subject.

Height, weight, BMI and OFC Z‐scores were calculated using a Microsoft Excel add‐in to access growth references based on the LMS method (Pan & Cole, 2012) using a reference European population (Cole, Freeman, & Preece, 1998).

a Refers to age of examination.

b Abnormal MRI findings include mildly enlarged lateral ventricles and several loci of irregular signal in the brain parenchyma above the tentorium, in the posterior white matter and along the ependyma.

c Also patient G001284 (Carss et al., 2017).

Our clinical and genetic studies in six affected individuals, including additional new clinical details for Patient 3, (Carss et al., 2017) take the total number of *SCAPER* syndrome patients described to date to 23. Although the extent for which clinical data is available for the previously reported patients is variable, our detailed clinical phenotyping allows a more comprehensive clinical comparison to be made with the previously reported cases, confirming the presence of a variable pattern of dysmorphic features associated with *SCAPER* syndrome. It is now clear that the cardinal clinical features of the disorder include mild/moderate ID and developmental delay particularly affecting speech and language and motor milestones. Hyperactivity appears to be a common feature, with some affected individuals receiving a formal diagnosis of ADHD. Early adult onset RP is also a key clinical finding, and the retinal phenotype appears remarkably consistent. In all individuals for whom we have data, progressive loss of night vision begins in first or second decade of life. Together with studies in mice demonstrating expression of SCAPER in multiple retinal layers, particularly in the retinal pigment epithelium and photoreceptor inner and outer segments, this supports a role for SCAPER in photoreceptor function and/or maintenance (Tatour et al., 2017).

Tapering fingers, brachydactyly and proximally placed thumbs, described in eight individuals from two consanguineous Bedouin families of the same tribe in southern Israel, were also identified as a consistent feature in the two Amish siblings, confirming the association of this feature with the *SCAPER* syndrome. Short stature and obesity were also a common feature amongst the affected Bedouin patients, and this constellation of clinical features including RP, obesity, short stature, ID, developmental delay, and brachydactyly has consequently led to a suggested diagnosis of BBS in these individuals. Although there is some overlap between the clinical features characteristic of ciliopathies and those seen in SCAPER syndrome, the Amish siblings (who are of normal height and weight for age) demonstrate that the digital, retinal, and cognitive abnormalities may occur independently of short stature and obesity. The other common primary features of BBS, including renal anomalies, postaxial polydactyly, hypogonadism (males), and genital abnormalities (females) have not been reported in association with *SCAPER* mutation (Forsythe & Beales, 2013). The dysmorphic facial features and inverted nipples, noted on examination of both Amish siblings, have not been previously noted in other individuals with *SCAPER* variants.

Recently, a single individual homozygous for a c.2023‐2A>G *SCAPER* variant presenting with nonsyndromic RP and no evidence of ID was reported in this journal (Jauregui et al., 2019). The same c.2023‐2A>G *SCAPER* gene variant has also been reported previously in association with RP, ADHD, and mild ID (Tatour et al., 2017) indicating the variability in the presence and severity of the extraocular features associated with the *SCAPER* syndrome (Table 1). However, this may also be accounted for by the difficulties in conclusively defining milder developmental delay in some situations, when more subtle clinical findings may not be identified if not specifically assessed. Conversely, associated ocular pathology may remain undetected or unrecognized in individuals with ID, as such individuals often have difficulty recognizing or articulating their visual symptoms. This highlights the importance of visual screening and ophthalmological assessment in these patients. Other common ocular features include cataracts (in particular posterior subcapsular cataracts, which are commonly associated with RP; (Pruett, 1983) and strabismus, with nystagmus and keratoconus noted in a single patient. The high incidence of cataracts, a potentially treatable cause of sight loss, again supports the case for screening in early childhood.

The allele frequency (~0.04) of the Ohio Amish *SCAPER* founder mutation suggests that, despite no previous reports, this disorder represents an underrecognized cause of RP and mild ID within this community. This further highlights the importance of careful clinical evaluation in children and adults with ID and enables targeted genetic testing for this *SCAPER* variant for Amish individuals with this clinical presentation. Together with our clinical review of all previously published patients, this study expands the molecular spectrum of disease‐causing *SCAPER* variants and enables a clearer delineation of the core (and variable) phenotypical features of *SCAPER* syndrome to be characterized. Our findings also highlight the importance of prompt visual screening and ophthalmic assessment in all individuals with *SCAPER*‐associated disease. Despite the increasing numbers of individuals identified with *SCAPER* syndrome, the precise functions of SCAPER in human growth and development are not fully understood. Further studies to elucidate the precise molecular and developmental roles of this molecule in human growth and skeletal, retinal, and brain development and function, will yield important insights into the clinical heterogeneity increasingly observed in *SCAPER*‐associated disease.

## AUTHOR CONTRIBUTIONS

J. F., G. A., S.L. contributed equally to this work. E. L. B., A .R. W., and A. H. C. contributed equally to this work.

## Supporting information


**Table S1** Ocular findings of all affected individuals with biallelic pathogenic SCAPER variants
