# Supplementary material for: Delineating the expanding phenotype associated with SCAPER gene mutation
Source: Am J Med Genet A. 2019 Jun 13;179(8):1665–71. doi: 10.1002/ajmg.a.61202 (PMC6772143; doi:10.1002/ajmg.a.61202)
Supplement: Supplementary file 1 — Table S1 Ocular findings of all affected individuals with biallelic pathogenic SCAPER variants [file AJMG-179-1665-s001.docx]

Table S1. Ocular findings of all affected individuals with biallelic pathogenic SCAPER variants

| **Table S1. Ocular findings of all affected individuals with biallelic pathogenic *SCAPER* variants** | | | | | | | |  |  |  |  |  |
| --- | --- | --- | --- | --- | --- | --- | --- | --- | --- | --- | --- | --- |
|  | **Presenting age (yrs)** | **Nyctalopia** | **🡫 visual fields** | **VA logMAR (Snellen)** | **Strabismus** | **Cataract (morphology)** | **Optic disc pallor/ atrophy** | **Retinal vessel attenuation** | **Retinal pigmentary changes** | **CME** | **Electrophysiology** | **Other ocular findings** |
| Najmabadi | NA | NA | NA | NA | NA | NA | NA | NA | NA | NA | NA | NA |
| Tatour (A:II:1) | Dec-13 | ✓ | NA | Mod/Sev 🡫 | NA | ✓ | ✓ | ✓ | ✓ | ✓ | Undetectable rod and cone responses | Secondary glaucoma |
| Tatour (A:II:2) | Dec-13 | ✓ | NA | Mod/Sev 🡫 | ✓ | No | ✓ | ✓ | ✓ | No | Undetectable rod and cone responses | Nil |
| Tatour (B:II:1) | 28 | ✓ | ✓ | Mod 🡫 | NA | ✓ (PSC) | ✓ | ✓ | ✓ | ✓ | Extinguished rod and cone responses | Nystagmus |
|  |  |  |  |  |  |  |  |  |  |  |  | High myopia |
| Tatour (C:II:4) | 15 | NA | ✓ | 🡫 | NA | NA | ✓ | ✓ | ✓ | No | Abolished | Nil |
| Hu (Family 166; 3 individuals) | NA | NA | NA | NA | NA | NA | NA | NA | NA | NA | NA | NA |
| Jauregui | 9 | ✓ | NA | RE: 0.3 (20/40) | NA | NA | ✓ | ✓ | ✓ | No | Undetectable rod, subnormal cone responses | Nil |
|  |  |  |  | LE: 0.1 (20/25) |  |  |  |  |  |  |  |  |
| Wormser (P1:V5) | 10 | ✓ | NA | RE: PL | ✓ | ✓ (PSC) | ✓ | ✓ | ✓ | NA | Extinguished rod and cone responses | Nil |
|  |  |  |  | LE: HM |  |  |  |  |  |  |  |  |
| Wormser (P1:V6) | 15 | ✓ | NA | RE: 0.3 (20/40) | ✓ | ✓ (PSC & nuclear, punctate) | ✓ | ✓ | ✓ | NA | Extinguished rod and cone responses | Nil |
|  |  |  |  | LE: 0.3 (20/40) |  |  |  |  |  |  |  |  |
| Wormser (P1:V7) | 13 | ✓ | NA | RE: 1.0 (20/200) | ✓ | ✓ (PSC) | ✓ | ✓ | ✓ | NA | NA | Nil |
|  |  |  |  | LE: 2.0 (20/400) |  |  |  |  |  |  |  |  |
| Wormser (P1:V8) | 7 | ✓ | NA | RE: 0.24 (20/33) | No | No | NA | NA | Suspected | NA | NA | Nil |
|  |  |  |  | LE: 0.24 (20/33) |  |  |  |  |  |  |  |  |
| Wormser (P2:III1) | 20 | ✓ | NA | RE: NPL | No | ✓ (mild cortical) | ✓ | ✓ | ✓ | ✓ | NA | Nil |
|  |  |  |  | LE: NPL |  |  |  |  |  |  |  |  |
| Wormser (P2:III2) | 28 | ✓ | NA | RE: HM 15cm | ✓ | ✓ (mild PSC) | ✓ | ✓ | ✓ | ✓ | NA | Nil |
|  |  |  |  | LE: PL |  |  |  |  |  |  |  |  |
| Wormser (P2:III7) | 25 | ✓ | NA | RE: 0.54 (20/70) | No | No | No | No | ✓ | NA | NA | Nil |
|  |  |  |  | LE: 0.54 (20/70) |  |  |  |  |  |  |  |  |
| Wormser (P2:IV1) | NA | Unable to assess | NA | Fixes and follows objects | No | No | ✓ | No | ✓ | NA | NA | Nil |
| **Patient 1** | 13 | ✓ | No | RE: 0.48 (20/60) | ✓ | No | No | No | No | No | NP | Bilateral meridonal amblyopia |
|  |  |  |  | LE: 0.48 (20/60) |  |  |  |  |  |  |  |  |
| **Patient 2** | NA | NA | NA | NA | NA | NA | NA | NA | NA | NA | NA | NA |
| **Patient 3*** | 14 | ✓ | ✓ | RE: 0.3 (20/40) | NA | No | ✓ | ✓ | ✓ | No | undetectable rod responses, | Keratoconus |
|  |  |  |  | LE: 0.3 (20/40) |  |  |  |  |  |  | pERG subnormal |  |
| **Patient 4** | 10 | ✓ | NA | RE: 0.2 (20/30) | NA | ✓ | ✓ | ✓ | ✓ | NA | Undetectable rod responses | Myopia |
|  |  |  |  | LE: 0.3 (20/40) |  |  |  |  |  |  |  |  |
| **Patient 5** | NA | ✓ | ✓ | NA | NA | NA | ✓ | ✓ | ✓ | ✓ | Severe 🡫 | Nil |
| **Patient 6** | 23 | Unable to definitively assess | ✓ | RE: 0.2 (20/30) | ✓ | ✓ | NA | ✓ | ✓ | ✓ | Undetectable rod, severely 🡫 | Nil |
|  |  |  |  | LE: 0.3 (20/40) |  | (nuclear) |  |  |  |  | cone responses |  |
| Total | - | 15/15 | 5/6 | - | 7/11 | 9/15 | 14/16 | 14/17 | 16/17 | 6/11 | 11/11 | - |
| Abbreviations: CME, cystoid macular edema; HM, detection of hand motion; LE, left eye; Mod, moderate; NA, not available; NPL; no perception of light; pERG, pattern ERG; PL, perception of light; PSC, posterior subcapsular cataract; RE, right eye; Sev, severe; VA, visual acuity. (✓) indicates presence of a feature in an affected subject | | | | | | | | | | | | |
| * Also patient G001284 (Carss *et al.* 2017) | | |  |  |  |  |  |  |  |  |  |  |
